# Perceived Disease Risk of Smoking, Barriers to Quitting, and Cessation Intervention Preferences by Sex Amongst Homeless Adult Concurrent Tobacco Product Users and Conventional Cigarette-Only Users

**DOI:** 10.3390/ijerph19063629

**Published:** 2022-03-18

**Authors:** Haleem A. Brown, Rachel D. Roberts, Tzuan A. Chen, Michael S. Businelle, Ezemenari M. Obasi, Darla E. Kendzor, Lorraine R. Reitzel

**Affiliations:** 1 Department of Psychological, Health, and Learning Sciences, College of Education, The University of Houston, 491 Farish Hall, Houston, TX 77204, USA; hbrown5@cougarnet.uh.edu (H.A.B.); rdrober3@cougarnet.uh.edu (R.D.R.); tchen3@central.uh.edu (T.A.C.); emobasi@central.uh.edu (E.M.O.); 2 HEALTH Research Institute, The University of Houston, 4349 Martin Luther King Blvd., Houston, TX 77204, USA; michael-businelle@ouhsc.edu; 3 TSET Health Promotion Research Center, The University of Oklahoma Health Sciences Center, Oklahoma City, OK 73104, USA; darla-kendzor@ouhsc.edu

**Keywords:** tobacco use, smoking, homeless, concurrent tobacco use, perceived disease risk, cessation preferences, sex differences, cessation medications

## Abstract

Adults experiencing homelessness smoke conventional cigarettes and engage in concurrent tobacco product use at very high rates; however, little is known about how use patterns, perceived disease risk, barriers to quitting smoking, and smoking cessation intervention preferences differ by sex in this group. Participants comprised a convenience sample of 626 adult conventional cigarette smokers experiencing homelessness. Participants self-reported their sex, smoking history, mental health and substance use diagnosis history, other concurrent tobacco product use (CU), disease risk perceptions, perceived barriers to quitting smoking, and preferences regarding tobacco cessation interventions via a computer-administered survey. CU rates were 58.1% amongst men and 45.3% amongst women smokers. In both sexes, CUs started smoking earlier (*p*-values < 0.001) and were more likely to have been diagnosed with a non-nicotine substance use disorder (*p*-values < 0.014) relative to cigarette-only users. Among men only, CUs were younger, smoked more cigarettes per day and were more likely to identify as non-Hispanic White (*p*-values < 0.003) than cigarette-only users. Additionally, male CUs reported a greater risk of developing ≥1 smoking-related disease if they did not quit for good; were more likely to endorse craving cigarettes, being around other smokers, habit, stress/mood swings, and coping with life stress as barriers for quitting smoking; and were less likely to prefer medications to quit smoking relative to male cigarette-only users (*p*-values < 0.04). On the other hand, female CUs reported a greater risk of developing ≥1 smoking-related disease even if they quit for good; were more likely to endorse stress/mood swings and coping with life stress as barriers for quitting smoking relative to female cigarette-only users (*p*-values < 0.05); and did not differentially prefer one cessation medication over another. Overall, findings confirm high rates of CU among both sexes, characterize those who may be more likely to be CUs, and reveal opportunities to educate men and women experiencing homeless on the benefits of evidence-based interventions for smoking cessation.

## 1. Introduction

There are 4.2% of adults who currently live in the United States (US) who will experience homelessness at some point in their lifetime [1]. According to the 2020 Annual Homeless Assessment Report, over 500,000 people experience homelessness in the US annually [2]. Approximately 70 to 80% of the adult homeless population in the US smokes conventional cigarettes [3,4,5,6,7], a rate much higher than that of housed adults. Likewise, tobacco-attributable mortality rates have been estimated to be 3- to 5-fold higher amongst adults experiencing homelessness relative to the domiciled population [8]. Moreover, not only are smoking rates high within this group, so too are rates of dual or poly-tobacco product use (hereafter referred to as concurrent use). For example, in one study, concurrent use rates among adult homeless smokers were 51.1% [9]; in another study, they were as high as 68% [10]. These rates far exceed the concurrent use of tobacco products amongst adult domiciled smokers, which is cited as approximately 7.9–10.6% [11,12]. Concurrent tobacco use confers additional health risks over and above conventional cigarette smoking; for example, increased oral or pharyngeal cancer risk due to enhanced exposure to carcinogenic toxins [13]. Additionally, adult concurrent tobacco users may also be less willing to quit smoking relative to adults who are cigarette-only smokers [14]. Consequently, it is critically important to motivate quit attempts and intervene to address tobacco use disorder in this group to mitigate the disparities they experience in associated morbidity and mortality.

In the domiciled US population, there are established differences in tobacco use and abstinence rates by sex. For example, national data support higher conventional cigarette smoking rates among domiciled men (~15.3%) than women (~12.7%) [15]. However, men may be more likely to achieve long-term abstinence from smoking relative to women [16]. Additionally, concurrent tobacco product use tends to be more common amongst men than women in the US [17]; for example, one national study on concurrent use reported that 1.6% of men were concurrent users of cigarettes and smokeless tobacco verses 0.3% of women [18]. These differences between the sexes have been attributed to both biological and psychosocial factors affecting tobacco use uptake, nicotine dependency, and smoking motives [19,20,21]. For example, women reported being more motivated to smoke to relieve negative emotional states and symptoms than men [22]. However, smoking in response to negative emotional states was associated with having more significant barriers to quitting smoking [22]. Another work suggested, through an experimental paradigm, that some men—in this case, men with significant depression—experienced greater craving and nicotine withdrawal than women; however, women overall were less likely than men to successfully abstain from smoking when asked [23]. Such differences may suggest the need for or utility of tailoring cessation interventions by sex—and perhaps other factors—for improved outcomes [24]. For example, extant literature suggests that cessation interventions for women include discussion of things potentially more relevant to that sex, such as understanding the benefits of practicing self-care and how an important form of self-care is quitting smoking [25,26]. However, although sex differences in smoking behaviors and concurrent tobacco product use have been studied extensively in domiciled samples, relatively little work has examined whether these patterns persist amongst adults experiencing homelessness, a marginalized group with disproportionately high conventional smoking and concurrent use rates. A better understanding of reasons for concurrent product use, perceived disease risk from smoking, barriers to quitting, and cessation intervention preferences by sex or by status as a smoker only versus a concurrent tobacco product user may be helpful to inform and adapt interventions directed toward tobacco-using men and women experiencing homelessness.

In conclusion, there have been few studies with individuals experiencing homelessness that have examined concurrent tobacco product use, and none have examined sex differences in reasons for concurrent use, perceptions of disease risk from smoking, barriers to quitting smoking, and preferences regarding cessation interventions. The purpose of the current study was to address these gaps in the literature in a large, pooled sample of homeless adult smokers. Better understanding the tobacco use behaviors and associated constructs among homeless smokers versus homeless smokers who also concurrently use other tobacco products, by sex, can help to inform effective approaches to cessation interventions within this vulnerable group. This is critically important given that an estimated 72% of adult homeless smokers make a quit attempt at least annually, with ~37% being willing to make a smoking quit attempt within the next 6 months [6].

## 2. Materials and Methods

### 2.1. Procedures

This was a secondary data analysis that combined data from two studies focused on the health and health risks of adults experiencing homelessness. The first study (Study 1) was conducted at a shelter in Dallas, Texas, and the second study (Study 2) was conducted within 6 agencies providing shelter and/or services to individuals experiencing homelessness in Oklahoma City, Oklahoma. Participants were recruited in both studies using flyers posted in the targeted settings. Potential participants were screened on site relative to the following inclusion criteria: aged 18 or over, currently receiving services (e.g., shelter, food, and counseling) at the targeted agencies, currently homeless, English speaking, and at least a 7th grade English literacy level (as assessed by the Rapid Estimate of Adult Literacy in Medicine-Short Form) [27]. Details regarding the study design and methods are referenced in other work [28,29,30,31,32]. Associated academic institutions provided approval for study conduct and informed consent was obtained from all participants.

Overall, a total of 394 (Study 1) and 610 (Study 2) participants enrolled. Participants responded to survey items that were administered on a laptop computer or tablet. The survey items were displayed visually and were read aloud to the participant via headphones. Remuneration for participation was provided ($20 department store gift card). Data were collected in 2013 (Study 1) and 2016 (Study 2). All participants in Study 1 were homeless by virtue of residing at the shelter, whereas 581 individuals from Study 2 endorsed current homelessness (i.e., self-identified as homeless and endorsed staying at a friend or family member’s house, homeless shelter, outside or on the street, hotel/motel, drug/alcohol treatment center, or other temporary location). The analytic sample was then narrowed to participants who endorsed being current conventional cigarette smokers (*n* = 299, Study 1; *n* = 457, Study 2), and then further reduced to those who were asked about concurrent tobacco product use (*n* = 177, Study 1; *n* = 449, Study 2).

### 2.2. Measures

#### 2.2.1. Participant Characteristics

Participant characteristics included sex, age, race/ethnicity, last month’s income, education, lifetime number of months homeless, and self-reported lifetime alcohol or non-nicotine comorbid substance use disorder diagnosis, and self-reported lifetime history of receipt of a severe mental illness diagnosis (i.e., depression, bipolar, schizophrenia or schizoaffective disorder).

#### 2.2.2. Cigarette Dependence

The self-reported age when participants started smoking, number of years smoked, and average number of cigarettes smoked per day (CPD) were assessed.

#### 2.2.3. Concurrent Tobacco Use

Concurrent users were conventional cigarette smokers endorsing use of a non-cigarette tobacco or nicotine product in the last 30 days. Options were: (a) snus, such as Camel or Marlboro snus; (b) roll-your-own cigarettes; (c) tobacco from a hookah or a waterpipe; (d) dissolvable tobacco products such as Ariva/Stonewall/Camel/Camel Orbs/Camel sticks; (e) electronic cigarettes or E-cigarettes (including battery-operated vape pens, e-pipes, e-cigars, personal vaporizers, or e-hookahs), such as Fin, NJOY, Blu, e-Go, and Vuse; (f) cigars; (g) little cigars/cigarillos/bidis; (h) chewing tobacco, dip, or snuff; and/or (i) other tobacco product (besides conventional cigarettes). Pictures of generation 1, 2, and 3 e-cigarettes accompanied question text.

#### 2.2.4. Concurrent Tobacco Use Frequency

Frequency of concurrent use in in an average week was assessed, with the following options: (a) every day; (b) 5 to 6 days; (c) 3 to 4 days; (d) 1 to 2 days, (e) less than 1 day; and (f) I don’t know. The number of times used on days in which concurrent products were used were also assessed with an item reading: “On the days you use [insert endorsed product], how many times a day do you use it?”

#### 2.2.5. Perceived Risk of Smoking

Perceived disease risk of smoking was assessed with 2 items. The first asked: “What are your chances of developing at least one smoking-related disease if you quit for good?” and the second asked: “What are your chances of developing at least one smoking-related disease if you do NOT quit for good?” Answer options for each item were in increments of 10 percentage points with descriptors ranging from “0%—I will DEFINITELY NOT develop”, to “50%—I have a 50/50 chance”, to “100% I will DEFINITELY develop.” For analyses, answers were converted to a 0–10 scale.

#### 2.2.6. Perceived Barriers to Quitting Smoking

Perceived barriers to quitting smoking were assessed with the following investigator-generated, face-valid, “check all that apply” item: “Which of these would be the hardest thing(s) about stopping smoking?” Answer options were: (a) craving cigarettes; (b) being around other smokers; (c) fear of weight gain; (d) habit; (e) stress/mood swings; (f) coping with life stress; and (g) avoiding friends who smoke.

#### 2.2.7. Cessation Intervention Preferences

Preferences for future quit attempts were assessed with 3 investigator-generated, face-valid items. The first item read: “Which of the following options would give you the best chance for quitting smoking?” Answer options were: (a) medications; (b) group counseling; (c) both medications and counseling; and (d) quitting “cold turkey”—without counseling or medications. The second item read: “Would you prefer to use tobacco cessation medications if you were to try to quit in the future?” Answer options were yes or no. The third item read: “If you were to try to quit smoking, which tobacco cessation medication would you prefer?” Answer options were: (a) Chantix/Varenicline; (b) Zyban/Wellbutrin; (c) nicotine patch; (d) nicotine gum; (e) nicotine nasal spray or other medication; and (f) no medications.

### 2.3. Data Analysis

The sample was stratified by sex prior to analysis. The prevalence of CU was calculated from the data. For concurrent users, the products used and frequency of use were reported using descriptive statistics. Next, differences between concurrent users and smokers only on participant characteristics, cigarette dependence, perceived risk of smoking, perceived barriers to quitting smoking, and cessation intervention preferences were examined using chi-square and t-tests depending on the variables of interest. Of the analyzable sample of 626 persons, missing data on any variable ranged from 0% to 7.35% with no patterns related to missingness. Significance was set at *p* < 0.05. All analyses were conducted using SAS 9.4 (SAS Institute, Cary, NC, USA).

## 3. Results

### 3.1. Sample Descriptives

Of the 626 adult conventional cigarette smokers, 32.1% (*n* = 201) were women and 54.0% (*n* = 338) were concurrent tobacco product users. Concurrent use rates were 58.1% amongst men and 45.3% amongst women smokers. In both sexes, concurrent users starting smoking earlier (men: 15.09 vs. 17.72 years old; women: 15.32 vs. 18.31 years old, *p*-values < 0.001) and were more likely to have been diagnosed with a non-nicotine substance use disorder (men: 40.89% vs. 25.84%, women: 42.86% vs. 26.36%, *p*-values < 0.014) relative to smokers only. Among men only, concurrent users were younger (43.03 vs. 46.45, *p* = 0.0023), smoked more cigarettes per day (11.02 vs. 8.97, *p* = 0.0095), and were more likely to identify as non-Hispanic White (55.74% vs. 38.42%, *p* < 0.0001) than smokers only (Table 1).

### 3.2. Concurrent Tobacco Use Frequency by Sex

Concurrent tobacco use varied by product. For both sexes, the most commonly used product was roll-your-own cigarettes, followed by cigars for men and electronic cigarettes for women, and little cigars/cigarillos/bidis for men and women. More than half of those who reported use of roll-your-own cigarettes endorsed use at least 3–4 days (men) or at least 1–2 days (women) a week. Both male and female concurrent users who reported electronic cigarette use used that product more frequently than any other type of concurrent tobacco product. Male and female concurrent users reported using 12.71 and 8.79 times, respectively, on days when electronic cigarettes were used (Table 2).

### 3.3. Perceived Risk of Smoking by Sex

Male concurrent tobacco product users reported a greater perceived risk of developing ≥1 smoking-related disease if they did not quit smoking (6.45 vs. 5.80, *p* = 0.0327) relative to male smokers only. On the other hand, compared to female smokers only, female concurrent users reported a greater perceived risk of developing ≥1 smoking-related disease *even if* they quit smoking (4.25 vs. 3.50, *p* = 0.0431) (Table 3).

### 3.4. Barriers to Quitting Smoking by Sex

Compared to male smokers only, male concurrent users were more likely to endorse craving cigarettes (68.57% vs. 58.76%, *p* = 0.0377), being around other smokers (55.92% vs. 40.11%, *p* = 0.0014), habit (54.69% vs. 37.85%, *p* = 0.0006), stress/mood swings (59.59% vs. 45.2%, *p* = 0.0034), and coping with life stress (50.61% vs. 35.03%, *p* = 0.0015) as barriers for quitting smoking. Female concurrent users were more likely to endorse stress/mood swings (76.92% vs. 59.63%, *p* = 0.0093) and coping with life stress (69.23% vs. 53.21%, *p* = 0.0093) as perceived barriers to quitting smoking relative to female smokers only (Table 3).

### 3.5. Cessation Intervention Preferences by Sex

Male concurrent users were less likely to prefer medications to quit smoking (30.61% vs. 40.68%, *p* = 0.0322) on their next quit attempt relative to male smokers only. Although there were not significant differences between concurrent users and smokers only regarding what would give them the best chance of quitting smoking, both sexes most endorsed “cold turkey” (vs. medications/counseling) as their best option for quitting. Finally, concurrent users did not differ from smokers only on preferred tobacco cessation medication (Table 3).

## 4. Discussion

The current study confirmed high rates of conventional cigarette smoking (~75% of the sample) and concurrent tobacco product use (54%) overall in this sample of adults experiencing homelessness from Dallas, TX US and Oklahoma City, OK US, with a concurrent tobacco product use prevalence of 58.1% in men and 45.3% in women. The of concurrent tobacco use among adult homeless smokers is ~6-fold higher than rates amongst adult domiciled smokers [11,12] and suggests a critical need to intervene to reduce tobacco use within settings serving adults experiencing homelessness. Overall, roll-your-own cigarettes was the most highly endorsed concurrent product used. The use of roll-your-own cigarettes is lower in the US in than in other countries [33,34]; however, its frequent use amongst individuals experiencing homelessness in this sample may reflect its cost-effectiveness relative to the purchase of conventional cigarettes that allows individuals with limited and potentially inconsistent financial means to satisfy nicotine addiction with either product. However, the high roll-your-own use rates are notable as at least one prior study found that young adult users were less motivated to quit smoking than those who smoke conventional cigarettes only [35]. Moreover, the use of this particular concurrent tobacco product, in conjunction with conventional cigarette smoking, has been shown to increase lung cancer risk [36].

An important main contribution of this paper to the literature, however, is the quantification of the perceived disease risk of smoking, barriers to quitting, and cessation intervention preferences by sex amongst homeless adult concurrent tobacco product users and conventional cigarette-only users. Specifically, perceived disease risk and barriers to quitting differed between conventional cigarette smokers only and concurrent tobacco product users in the male and female subsamples, respectively, whereas differences in cessation intervention preferences only emerged between male concurrent tobacco product users and their conventional cigarette-only using counterparts. Together, results provide initial information that may guide smoking cessation intervention content and emphasis for homeless men and women for those who do and do not concurrently use other tobacco products in addition to conventional cigarettes, as explicated more fully in the paragraphs below. This is important given that almost three-quarters of adult homeless smokers try to quit each year, with over one-third of smokers being willing to do so within the upcoming 6 months [6], often unassisted by health professionals [37]. Moreover, despite the increased health risks associated with dual or poly-tobacco product use [10], the literature suggests adult concurrent users may be less likely to quit smoking and have lower self-efficacy about quitting relative to adults who were smokers only [38], highlighting the need to know more about the drivers of concurrent use in this group and potential barriers to smoking cessation.

Both male and female concurrent users perceived greater risk of disease relative to their cigarette-only smoking counterparts. However, amongst men, these differences emerged regarding perceived risk if they *did not* quit whereas amongst women, they reflected perceived disease risk only *if they quit*. Additionally, despite relatively similar conventional cigarette smoking consumption rates (between 9 and 11 cigarettes per day on average), women endorsed greater perceived disease risk levels even if they were to quit relative to men; this was especially evident amongst concurrent tobacco products users (4.25 vs. 3.38 on a 10-point scale for concurrent users and 3.50 vs. 3.23 for cigarette-only smokers). These data suggest that female concurrent users may have a more fatalistic attitude about quitting and subsequent disease risk relative to men that might undervalue the benefits of smoking cessation to their health. Thus, results suggest that pro-cessation messaging and intervention content for female concurrent users should include corrective messaging about the health benefits of smoking cessation. On the other hand, the divergence between male concurrent users and smokers only on perceived disease risk if they did not quit smoking for good (6.34 vs. 5.80 on a 10-point scale) may be something that can be capitalized upon in smoking cessation interventions for male concurrent tobacco users to build motivation for a quit attempt through a reduction in smoking-related disease risk.

The examination of barriers to quitting smoking indicated that male concurrent users differed from smokers only in their elevated endorsement of cigarette cravings, being around other smokers, habit, stress/mood swings, and coping with stress as barriers to smoking cessation. These results suggest multi-dimensional/diverse barriers to quitting that would need to be part of cessation interventions for men and especially interventions for male concurrent users who endorse these barriers at relatively elevated rates versus cigarette-smokers only. The greater endorsement of habit and being around other smokers as barriers for male concurrent tobacco users may suggest the utility of tobacco-free shelter policy implementation in these settings that could reduce the salience of cues to use in/around shelter or homeless-serving agency entries, exits, and common living/socializing spaces. Amongst women, concurrent users endorsed greater stress/mood swings and coping with life stress relative to smokers only, suggesting the primary importance of addressing alternate and healthy coping methods for stress and mood swings within cessation interventions. This is consistent with a previous study in which women endorsed smoking to relieve negative affect states and symptoms, which also contributed to more severe quit problems, and more barriers to cessation intervention [22]. Additionally of note was that women endorsed several barriers to quitting smoking in greater proportions than their male counterparts. Specifically, smoking-only women endorsed every barrier assessed at greater proportions than men; concurrent tobacco product-using women endorsed craving cigarettes, being around other smokers, fear of weight gain, stress/mood swings, and coping with life stress—all but two barriers to quitting—in greater proportions than their male counterparts. Overall, results may suggest that cessation interventions for women experiencing homelessness may need to spend more time than those designed for men to explore and address the diverse barriers to quitting that women experience. Likewise, more time may need to be spent with women in building motivation to quit (and to persevere through quit attempts) despite these barriers. Our results are consistent with a previous study supporting the importance of tailoring concurrent tobacco use cessation interventions for women experiencing homelessness [39].

Finally, the field is clear about evidence-based interventions for tobacco use cessation. For example, research on smoking cessation indicates that medications, and particularly medications used in combination with one another, when combined with brief counseling, especially as delivered in every health care encounter, are effective in assisting a quit attempt [40]. This suggests that quitting “cold turkey” is less likely to lead to cessation success. In the present study, there were several notable patterns regarding cessation treatment preferences that failed to align with the extant evidence base on effective tobacco cessation interventions. First, male concurrent tobacco product users were significantly less likely than male smokers only to endorse a preference to use tobacco cessation medications/nicotine replacement therapies (NRT) during their next quit attempt. Considering that concurrent tobacco users may be more dependent on tobacco given their dual and poly-product use, medications/NRT may be particularly crucial to quit success for these individuals. Moreover, prior research suggests that the provision of NRT, particularly when provided free of cost, may increase motivation to seek other cessation resources, such as Quitline counseling [41]. Given the known benefits of the use of medications such as bupropion SR, nicotine gum, nicotine inhaler, nicotine lozenge, nicotine nasal spray, nicotine patch, and varenicline in assisting successful quit attempts for any tobacco user [42], the relatively low endorsement of preferring a medicinal intervention for cessation (between 30.61 and 40.68% of men and 35.16.and 36.70% of women) is alarming. This result fits with high rates of endorsement that the best chance of quitting would be to do so “cold turkey” (endorsed by 42.98–51.51% of men and 39.81–41.76% of women) and a preference for “no [preferred medicinal] aids” (endorsed by 25.71–31.64% of men and 20.88–21.10% of women). Together, these data suggest the importance of educating homeless tobacco users about evidence-based interventions for cessation if the assumption is that a lack of knowledge underlies these preferences. However, it is also possible that a perceived lack of ready access to such care may have influenced results. For example, although the Quitline in Texas offers free NRT (2 weeks of the nicotine patch, gum or lozenge) to eligible smokers, a residential address is needed for delivery and multiple shipments to the same address (e.g., a shelter) may impede receipt. In fact, tobacco use disorder amongst individuals experiencing homelessness has been characterized as a “neglected addition” in health care settings. In one study, the proportion of smoking-attributable deaths in a cohort of homeless adults in Boston rivaled that of alcohol and drug-related deaths [8,37]. Similarly, another Boston-based study of adults experiencing homelessness indicated that approximately 33% of all cancer deaths were smoking attributable [43]. Thus, it is important to contextualize our results about participants’ treatment preferences within the overarching standard of care for tobacco use amongst adults experiencing homeless, which may be quite limited, and which may differ by state, insurance coverage, access to health care, competing medical needs, etc. Nevertheless, additional research, perhaps of a qualitative nature, is needed to better understand the prior quit experiences amongst homeless tobacco users and their rationale for endorsements of cessation intervention preferences, particularly as they may diverge from known evidence-based interventions such as combining counseling with medication/NRT use [42]. However, it is important to note a general lack of difference between concurrent users and smokers only of each sex on preferred tobacco cessation medication, with the greatest endorsement in each group for the nicotine patch. This information suggests that homeless serving agencies can assist their tobacco-using stakeholders in procuring over-the-counter cessation medication such as the patch to address this preference for quitting, while providing referrals to prescribers for the 10–20% who might prefer Chantix prescription. Finally, the overall low endorsement of a preference for group counseling (between 3.30 and 7.41% of the subsamples) requires further research, as it is unclear if this reflects an aversion to group counseling (which might be a preferable approach in limited resource settings), counseling in general, or counseling without medications. Qualitative research may help to inform the reasons for these preferences, and thus highlight potential strategies to make group cessation counseling more attractive to this population (e.g., sex-specific group counseling, coupling counseling and incentives for counseling session attendance) [44].

There were several limitations to this study that warrant consideration. First, although we combined two datasets and had a large sample size, the underlying data collections comprised convenience samples. Moreover, data were collected from a total of seven homeless-serving locations/shelters but in only two cities in the southern US. Thus, generalizability of findings may be limited and may not represent adults experiencing homelessness in other regions or cities in the US, wherein adults may have different access to tobacco products and cessation intervention programs. However, the Oklahoma City data collection captured approximately 38% of the underlying local population of adults experiencing homelessness [45] and the Dallas data collection captured approximately three-quarters of the shelter residents [29], with racial and sex distributions grossly representative of those found in a point-in-time count of the homeless population in the local area [46]. However, it is important to note that point-in-time counts are merely estimates and that individuals experiencing homelessness may move within and across geographic areas; thus, these estimates likely do not capture all homeless adults in the true underlying population of one city or the other. Another threat to the generalizability of these results is that the samples were English speaking and literate at the 7th grade level; therefore, results may not represent those who speak another language or who have lower literacy. Additionally, the Dallas shelter did not admit pregnant women or adults with children in tow and did not include those who were unsheltered at the time of data collection. Thus, results from that proportion of the study sample are not representative of all adults experiencing homelessness. In the Oklahoma sample, while the included participants all self-reported homelessness, some may have been staying in a shelter setting whereas other may have been living unsheltered. Thus, the Oklahoma sample may have included both sheltered and unsheltered homeless adults and differs from the Dallas sample in that respect. However, both samples were derived from the target group of interest, adults experiencing homelessness (who smoked cigarettes). Differences between sheltered and unsheltered adults on the associations of interest in this study could not be assessed with the information gathered and should be the focus of future work. Finally, our data relied on self-report and retrospective recall, which can be biased. Future work in this area can be improved with objective measures such as medical records and by using validated scales that can enable comparison with other samples and populations.

## 5. Conclusions

In conclusion, results from this study confirm high rates of concurrent tobacco use among both sexes, characterize the nature of concurrent tobacco product users and product use prevalence for each sex, and reveal opportunities to educate men and women experiencing homelessness on the benefits of evidence-based interventions for smoking cessation to increase the likelihood of successful quit attempts. Results of this study elucidated perceived disease risk and perceived barriers to quitting smoking in concurrent tobacco product-using (vs. cigarette-only smoking) men and women that may support a need for tailored interventions by sex in these settings. In particular, interventions among women in general and concurrent tobacco product-using women in particular may need to focus on reducing fatalism and building motivation for quitting. For both sexes, education about evidence-based methods for quitting—coupled with accessibility of interventions—is advisable based on high endorsement of “cold turkey” as a preferred cessation approach (versus receiving counseling and/or medications). Practitioners and shelter administrators should aim to build capacity to provide cessation intervention education and resources locally to facilitate quit attempts, including with NRT or other evidence-based medications. Future work, potentially of a qualitative nature, may help to further clarify quantitative endorsements and provide additional insights into how to maximize quitting success within this marginalized group at significant risk of tobacco-related morbidity and mortality.

## Figures and Tables

**Table 1:** Sample Descriptives and Differences by Product Use Status (*N* = 626, 32.1% women).

**Men**	**Concurrent Users (*n* = 247)**	**Smokers Only (*n* = 178)**	**t or *Χ*^2^ Value**	***p*-Value**
	**M (± SD) or % [*n*]**			
**Participant Characteristics**				
Age	43.03 (11.59)	46.45 (10.84)	3.07	0.0023
Race/Ethnicity			29.81	<0.0001
Non-Hispanic White	55.74 [136]	38.42 [68]		
Non-Hispanic Black	24.18 [59]	49.15 [87]		
Hispanic	4.51 [11]	3.95 [7]		
Non-Hispanic Native American/Alaska Native	8.61 [21]	3.39 [6]		
All Other non-Hispanic races	6.97 [17]	5.08 [9]		
Last Month’s Income (in USD) *	386.85 (675.91)	308.69 (504.47)	−1.31	0.1913
Education (in years)	11.66 (1.92)	11.83 (1.65)	0.95	0.3409
Lifetime Months Homeless	44.3 (52.32)	46.79 (58.94)	0.46	0.6473
Severe Mental Illness Dx.			3.65	0.0561
No	36.84 [91]	46.07 [82]		
Yes	63.16 [156]	53.93 [96]		
Substance Use Disorder Dx.			10.35	0.0013
No	59.11 [146]	74.16 [132]		
Yes	40.89 [101]	25.84 [46]		
**Cigarette Dependence**				
Years Smoked	23.11 (12.61)	22.67 (12.94)	−0.35	0.7269
Age Started Smoking	15.09 (4.06)	17.72 (7.78)	4.09	<0.0001
Cigarettes Smoked per Day	11.02 (8.04)	8.97 (7.88)	−2.60	0.0095
**Women**	**Concurrent Users (*n* = 91)**	**Smokers Only (*n* = 110)**	**t or *Χ*^2^ Value**	***p*-Value**
	**M (± SD) or % [*n*]**			
**Participant Characteristics**				
Age	40.55 (11.62)	43.06 (11.19)	1.54	0.1253
Race/Ethnicity			7.6083	0.1070
Non-Hispanic White	55.56 [50]	50.91 [56]		
Non-Hispanic Black	15.56 [14]	26.36 [29]		
Hispanic	6.67 [6]	6.36 [7]		
Non-Hispanic Native American/Alaska Native	17.78 [16]	8.18 [9]		
All Other non-Hispanic races	4.44 [4]	8.18 [9]		
Last Month’s Income (in USD) *	329.38 (561.96)	335.77 (567.75)	0.08	0.9384
Education (in years)	11.51 (2.04)	11.93 (1.92)	1.51	0.1329
Lifetime Months Homeless	37.57 (40.75)	31.99 (44.59)	−0.91	0.3625
Severe Mental Illness Dx.			0.3649	0.5458
No	25.27 [23]	29.09 [32]		
Yes	74.73 [68]	70.91 [78]		
Substance Use Disorder Dx.			6.052	0.0139
No	57.14 [52]	73.64 [81]		
Yes	42.86 [39]	26.36 [29]		
**Cigarette Dependence**				
Years Smoked	21.08 (11.53)	18.58 (12.16)	−1.48	0.140
Age Started Smoking	15.32 (5.04)	18.31 (7.48)	3.35	0.001
Cigarettes Smoked per Day	11.78 (8.09)	9.69 (8.46)	−1.78	0.0772

Note. **Bolded** headers represent major topical areas. M = Mean; SD = Standard Deviation; % = percent; *n* = sample size; USD = US dollars. * = self-reported monthly income was not adjusted to reflect cost of living differences in Dallas versus Oklahoma City, which are estimated to be −4.4% per salary.com. Overall, 43.97% of the sample reported no monthly income from any source and 89.94% were not employed.

**Table 2:** Frequency of Endorsed Concurrent Nicotine and Tobacco Product Use (concurrent users *N* = 338).

	Product Use	Snus	Roll Your Own Cigarettes	Tobacco from a Hookah or Waterpipe	Dissolvable Tobacco Products	Electronic Cigarettes	Cigars	Little Cigars/Cigarillos/Bidis	Chewing Tobacco, Dip, or Snuff	Other Tobacco Products
		M (± SD) or % [*n*]								
**Men**	Total Users * (n)	29	148	6	4	77	108	88	57	22
*N* = 247	**Use Frequency**									
	Everyday	20.69 [6]	29.05 [43]	16.67 [1]	0.00 [0]	25.97 [20]	15.74 [17]	17.05 [15]	23.08 [57]	31.82 [7]
	5–6 days a week	3.45 [1]	10.81 [16]	16.67 [1]	25 [1]	10.39 [8]	10.19 [11]	3.41 [3]	24.56 [14]	0.00 [0]
	3–4 days a week	17.24 [5]	14.19 [21]	16.67 [1]	0.00 [0]	7.79 [6]	16.67 [18]	21.59 [19]	12.28 [7]	22.73 [5]
	1–2 days a week	13.79 [4]	21.62 [32]	0.00 [0]	25 [1]	12.99 [10]	29.63 [32]	26.14 [23]	12.28 [7]	18.18 [4]
	Less than 1 day a week	37.93 [11]	24.32 [36]	50 [3]	50 [2]	41.56 [32]	27.78 [30]	31.82 [28]	21.05 [12]	27.27 [6]
	Missing	6.9 [2]	0.00 [0]	0.00 [0]	0.00 [0]	1.3 [1]	0.00 [0]	0.00 [0]	29.82 [17]	0.00 [0]
	# times used daily when used	5.07 (6.66)	10.08 (8.92)	5.5 (5.24)	2 (1.41)	12.71 (21.66)	3.55 (3.14)	5.92 (7.43)	5.04 (4.85)	7.36 (6.71)
**Women**	Total Users * (n)	6	58	3	3	48	21	25	2	6
*N* = 91	**Use Frequency**									
	Everyday	16.67 [1]	17.24 [10]	0.00 [0]	0.00 [0]	18.75 [9]	4.76 [1]	4 [1]	0.00 [0]	16.67 [1]
	5–6 days a week	0.00 [0]	3.45 [2]	0.00 [0]	0.00 [0]	18.75 [9]	14.29 [3]	8 [2]	0.00 [0]	16.67 [1]
	3–4 days a week	0.00 [0]	13.79 [8]	0.00 [0]	33.33 [1]	8.33 [4]	14.29 [3]	16 [4]	50 [1]	0.00 [0]
	1–2 days a week	33.33 [2]	22.41 [13]	0.00 [0]	0.00 [0]	27.08 [13]	23.81 [5]	32 [8]	0.00 [0]	16.67 [1]
	Less than 1 day a week	50 [3]	43.1 [25]	100 [3]	66.67 [2]	27.08 [13]	42.86 [9]	40 [10]	50 [1]	50 [3]
	Missing	0.00 [0]	0.00 [0]	0.00 [0]	0.00 [0]	0.00 [0]	0.00 [0]	0.00 [0]	0.00 [0]	0.00 [0]
	# times used daily when used	1.83 (1.17)	6.21 (5.73)	1.33 (0.58)	3.33 (3.21)	8.79 (11.38)	3.05 (2.11)	4.28 (4.19)	2 (1.41)	7 (6.66)

Note. **Bolded** headers represent major topical areas. M = Mean; SD = Standard Deviation; % = percent; *n* = sample size. * = because concurrent tobacco product users could endorse use of >1 concurrent product, the total users are necessarily more than the number of men or women who provided data.

**Table 3:** Smoking Risk Perceptions, Barriers to Quitting Smoking, and Cessation Intervention Preferences by Sex (*N* = 626, 32.1% women).

Men	Concurrent Users (*n* = 247)	Smokers Only (*n* = 178)	t or *Χ*^2^ Value	*p*-Value
	M (± SD) or % [*n*]			
**Perceived risk of smoking**				
Chances of developing at least one smoking-related disease if you quit for good?	3.38 (2.69)	3.23 (2.68)	−0.60	0.5517
Chances of developing at least one smoking-related disease if you do NOT quit for good?	6.45 (3.08)	5.80 (3.04)	−2.14	0.0327
**Perceived barriers to quitting smoking**				
Craving cigarettes	68.57 [168]	58.76 [104]	4.32	0.0377
Being around other smokers	55.92 [137]	40.11 [71]	10.27	0.0014
Fear of weight gain	13.06 [32]	9.60 [17]	1.20	0.2740
Habit	54.69 [134]	37.85 [67]	11.68	0.0006
Stress/mood swings	59.59 [146]	45.20 [82]	8.56	0.0034
Coping with life stress	50.61 [124]	35.03 [62]	10.12	0.0015
Avoiding friends who smoke	27.76 [68]	21.47 [38]	2.16	0.1417
**Best chance for quitting smoking**			7.49	0.0579
Medications	30.17 [73]	19.32 [34]		
Group counseling	3.31 [8]	5.68 [10]		
Both medications and counseling	23.55 [57]	23.86 [42]		
Quitting “cold turkey”—without counseling or medications	42.98 [104]	51.14 [90]		
**Prefer to use tobacco cessation medications (next quit attempt)**			4.59	0.0322
Yes	30.61 [75]	40.68 [72]		
No	69.39 [170]	59.32 [105]		
**Preferred tobacco cessation medication**			4.78	0.4428
Chantix/Varenicline	15.10 [37]	11.30 [20]		
Zyban/Wellbutrin	8.57 [21]	6.78 [12]		
The nicotine patch	27.76 [68]	31.07 [55]		
The nicotine gum or lozenge or nasal spray	16.73 [41]	15.82 [28]		
Other medications	6.12 [15]	3.39 [6]		
No aids	25.71 [63]	31.64 [56]		
**Women**	**Concurrent Users (*n* = 91)**	**Smokers only (*n* = 110)**	**t or *Χ*^2^ Value**	***p*-Value**
	**M (± SD) or % [n]**			
**Perceived risk of smoking**				
Chances of developing at least one smoking-related disease if you quit for good?	4.25 (2.55)	3.50 (2.62)	−2.04	0.0431
Chances of developing at least one smoking-related disease if you do NOT quit for good?	6.36 (2.93)	6.06 (3.30)	−0.67	0.504
**Perceived barriers of quitting smoking**				
Craving cigarettes	71.43 [65]	61.47 [67]	2.19	0.1387
Being around other smokers	60.44 [55]	55.05 [60]	0.59	0.4423
Fear of weight gain	38.46 [35]	31.19 [34]	1.16	0.2815
Habit	52.75 [48]	53.21 [58]	<0.01	0.9478
Stress/mood swings	76.92 [70]	59.63 [65]	6.76	0.0093
Coping with life stress	69.23 [63]	53.21 [58]	6.76	0.0093
Avoiding friends who smoke	27.47 [25]	23.85 [26]	0.34	0.5587
**Best chance for quitting smoking**			1.60	0.6593
Medications	25.27 [23]	24.07 [26]		
Group counseling	3.30 [3]	7.41 [8]		
Both medications and counseling	29.67 [27]	28.70 [31]		
Quitting “cold turkey”—without counseling or medications	41.76 [38]	39.81 [43]		
**Prefer to use tobacco cessation medications (next quit attempt)**			0.05	0.8221
Yes	35.16 [32]	36.70 [40]		
No	64.84 [59]	63.30 [69]		
**Preferred tobacco cessation medication**			6.93	0.2256
Chantix/Varenicline	18.68 [17]	19.27 [21]		
Zyban/Wellbutrin	5.49 [5]	14.68 [16]		
The nicotine patch	30.77 [28]	29.36 [32]		
The nicotine gum or lozenge or nasal spray	13.19 [12]	11.01 [12]		
Other medications	10.99 [10]	4.59 [5]		
No aids	20.88 [19]	21.10 [23]		

Note. **Bolded** headers represent major topical areas. M = Mean; SD = Standard Deviation; % = percent; *n* = sample size.

## Data Availability

The data presented in this study are available on request from the corresponding author. The data are not publicly available due to ethical restrictions based on informed consent agreements.
